# Spatial-Temporal Movements of Free Ranging Pigs at the Wildlife-Livestock Interface of Murchison Falls National Park, Uganda: Potential of Disease Control at a Local Scale

**DOI:** 10.3389/fvets.2021.689377

**Published:** 2021-09-23

**Authors:** Ariane Payne, Peter Ogweng, Karl Ståhl, Charles Masembe, Ferran Jori

**Affiliations:** ^1^Department of Zoology, Entomology and Fisheries Sciences, Makerere University, Kampala, Uganda; ^2^National Veterinary Institute, SVA, Uppsala, Sweden; ^3^CIRAD, UMR Animal, Santé, Territoires, Risque et Ecosystèmes (ASTRE), Montpellier, France; ^4^UMR ASTRE, University of Montpellier, CIRAD, INRAE, Montpellier, France; ^5^Department of Zoology and Entomology, University of Pretoria, Pretoria, South Africa

**Keywords:** pig farming, pig diseases, GPS harness, home range, homestead, biosecurity, African swine fever

## Abstract

In many Ugandan rural communities, pigs are generally kept under traditional smallholder systems without basic biosecurity measures in place. In some instances, these systems are at the livestock-wildlife interface, as it is the case in Nwoya district, which is bordered by Murchison Falls National Park (MFNP). This pig system has potential for the maintenance and transmission of pathogens like African swine fever (ASF) between different herds, and also with wild pigs (warthogs and bushpigs). In this paper, we describe the spatial and temporal pattern of the movements of free ranging domestic pigs in a rural setting in Northern Uganda where ASF is endemic. We also determine their use of habitat to highlight the potential interaction hotspots between domestic pigs and between domestic and wild pig populations. We fitted 10 free-ranging domestic pigs owned by different homesteads with GPS harnesses during rainy and dry seasons. The pig home range, daily distance, activity pattern and habitat use were calculated. Our results show that the maximum area covered (MCP 100%) by the pigs varied between 35,965 and 475,077 m^2^. The core area varied from 1,317 to 50,769 m^2^. The pigs' home ranges were significantly bigger during the dry season than during the rainy season (Wilcoxon test, *W* = 22, *p* = 0.04). The mean full day (24 h) distance was longer in the dry season than in the rainy season (Student test, *t* = 2.7, *p* = 0.03). The pigs were mostly located within their own homestead, but they also used other homesteads, grass and crop fields. This study highlights that free-ranging domestic pigs may cover a wide area, especially during the dry season. Interestingly, the home range of pigs from different herds may overlap with areas used by wild pigs which share crops and other resources in this area. This study provides insights into a better understanding of the potential for spread of diseases such as ASF at small-scale and can be used to raise awareness of such risks and to better target implementation of preventive measures.

## Introduction

In East Africa, pig production has almost doubled in the last 10 years reaching over 12.5 million heads in 2014 (1). It has become a source of income for resource-poor farmers as pigs can be reared with low investments but have a high and fast productivity and a high feed conversion efficiency (2). Pigs are kept under a wide variety of farming systems, ranging from large-scale intensive and integrated systems to traditional smallholder systems where pigs are reared in free-ranging conditions, tethered or confined in locally built pigsties (2–5). This is the case in Uganda, where traditional farming systems prevails and where the development of the pig sector has been increasing since early 2000 (6, 7). The pig population in Uganda by 2016 stood at 4 million, according to Uganda Bureau of Statistics (8).

Under the free-ranging rearing system, pigs roam freely searching for food waste, scavenging or feeding on crops residues, reducing the cost and the labor of feeding and housing. This practice is often restricted to the dry season when crops have been harvested, whereas pigs are tethered or confined in small pens during the rainy season to prevent them from damaging the growing crops. However, this system albeit more affordable, hardly enables to meet nutritional requirements for pig growth and results in a low profitability. Furthermore, it exposes pigs to accidents, predation, theft and disease transmission since basic biosecurity measures are rarely implemented. Disease transmission may occur through direct or indirect contact with other wild or domestic animals or through contact with contaminated products or fomites. Some of the pathogens infecting pigs may raise public health issues and are considered by farmers as a main production constraint (2–6, 9, 10).

African swine fever (ASF), an infectious disease caused by ASF virus (ASFV), is considered a major limiting factor for the development of the pig farming in Africa (5, 11). This haemorrhagic, contagious and typically very lethal disease of pigs and Eurasian wild boar has neither treatment nor vaccine. In the East African context, ASFV can infect both domestic pigs and different species of wild suids such as warthogs (*Phacochoerus spp)* and bushpigs (*Potamocheorus spp*.) and soft ticks within the genus *Ornithodoros*. However, in Africa, only domestic pigs show clinical symptoms. Depending on the presence and overlap between these different hosts, the virus can circulate within a domestic cycle, involving domestic pigs and, in some cases, soft ticks and/or within a sylvatic cycle, involving warthogs, soft ticks and potentially bushpigs. The transmission can occur through direct or indirect contact, via infected carcasses, swill or fomites or tick bites. It is acknowledged that direct contact between pigs and movement of infected pigs and pig products represents the main way of dissemination of the virus (12–14). Nevertheless, in presence of an interface where wild and domestic hosts may interact, the domestic and sylvatic cycles can be connected and wild pigs may be a source of infection for domestic pigs, although the route of transmission remains poorly understood (12, 15).

ASF is endemic in Uganda and is considered the most fatal disease in pigs. The surveillance process starts with the farmer, who upon suspecting ASF, reports to nearest animal health worker or local authority. This is followed by a reporting chain going from the district veterinary officer to the commissioner of animal health (CAH) who dispatches a team from National Animal Disease Diagnostic and Epidemiological Center to undertake disease investigation and confirm or infirm the outbreak. Upon confirmation of the disease, CAH then informs OIE (World Organization for Animal Health). Outbreaks occur regularly with a peak often described during the dry season (5, 11, 16, 17). For instance, using report-driven investigations in 43 villages located in Gulu district, 211 outbreaks (the unit being the household) were reported between 2011 and 2014 (18). The occurrence of the sylvatic cycle has also been confirmed in Uganda, although the current importance is unknown (13, 19). Furthermore, in some areas, free-ranging domestic pigs coexist with warthogs and bushpigs, giving opportunity for the virus to circulate among and between the three species (16). Previous studies carried out in Uganda found that ASF outbreaks were associated with free-ranging pigs, small-scale farms, presence of warthogs burrows in the vicinity and the dry season (5, 16, 20–22). Moreover, the estimation of the basic reproduction number (R_0_) of ASF in small holder free-range pig production system in northern Uganda ranged between 1.58 and 3.24, depending on the method (23), indicating that free-ranging system prompt maintenance and between herd transmission of ASFV. These results suggest that, in Uganda, free-ranging pigs might be exposed to ASFV through contact with pigs from other herds and with infected material and potentially through interaction with wild hosts. Previous studies by our research in the same study area group suggest that indirect interactions between domestic pigs and wild pigs are frequent, and that they may pose an opportunity for disease transmission, particularly during the dry season and at water sources or crop fields (22, 24).

However, fine-scale studies describing how free-ranging pigs may interact with other free-ranging pigs and with wild pigs have been lacking. Improving the knowledge on the spatial behavior of the free-ranging pigs is thus needed to assess more precisely how this husbandry practice may contribute to the spread of ASF at a local scale in an area where ASFV is circulating. Moreover, such knowledge can help to better target preventive measures aimed at mitigating the spread of diseases such as ASF in traditional pig farming systems lacking basic biosecurity. In this paper, we describe the spatial and temporal pattern of the movements of free ranging domestic pigs and determine their use of habitat. Our results aimed to provide an idea of the average home range and activity covered by domestic pigs living in proximity of the boundaries of Murchison Falls National Park (MFNP). This information, combined with results from previous studies implemented in the area and documenting wild pigs incursion into farmland (22, 24), provide insight into the potential interactions occurring between domestic pigs and wild pigs at the interface of a large protected area in Northern Uganda.

## Materials and Methods

### Study Site

**Figure 1:**
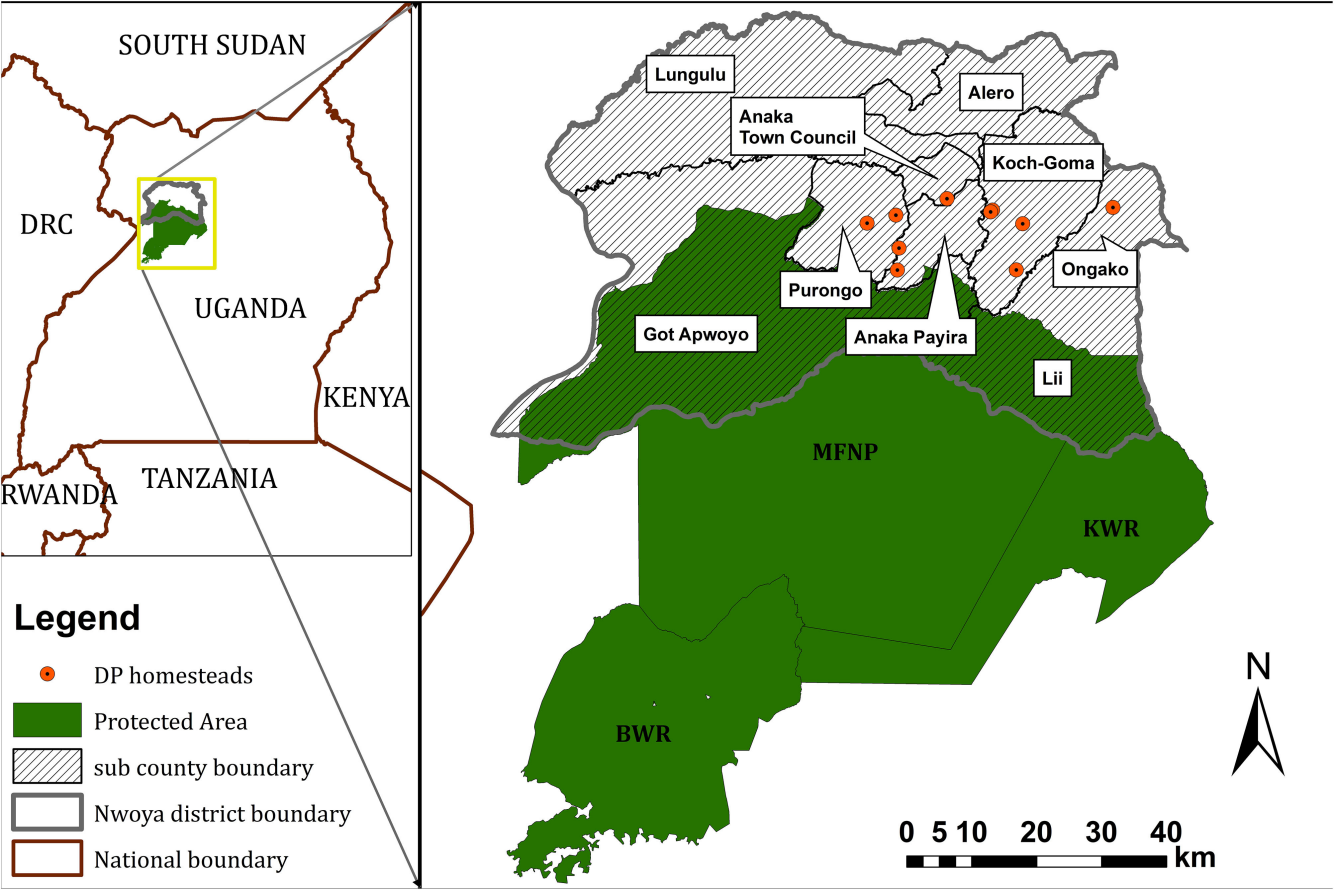
Location of the study site showing the homesteads from which domestic pigs (DP) were collared within Nwoya district and the Murchison Falls Conservation area composed of Murchison Falls National Park (MFNP), Karuma Falls Wildlife Reserve (KFWR) and Bugungu Wildlife Reserve (BWR).

The study area was located in North Western Uganda, in Nwoya district (total human population: 138,500; area: 4,736 km^2^), an administrative unit in the Acholi subregion (Figure 1). It comprised the northern boundary of MFNP and the adjacent rural communities at a maximum distance of 25 km from the park boundary. The vegetation consists of mostly savannah and the region is covered by a mixture of grassland and farmland interspersed by small woods. The major crops cultivated in Nwoya district are groundnuts, beans, maize, rice, cassava and sesame (24, 25). The major livestock species in Nwoya district are cattle, goats, sheep and pigs (22). The population of pigs in Nwoya stands at 12,800 as of 2019 according to the official figures of the Nwoya District Production office. Most farmers are smallholders, keeping between 1 and 4 pigs on free range during the dry season and most often tethering during the wet season with the local and cross breeds most preferred although few farmers also keep exotic breeds (especially Camborough and Large white). The climate is tropical with a rainy season from April through November and a dry season from December to March. Warthogs and bushpigs are widespread in the unfenced national park that borders the study area but they are also seen up to 25 km from such border into the farmland area. Based on farmers sightings reports in the same district, Kukielka et al. (22) assessed a density of individuals/km^2^ ranging from 0 to 10 for warthogs and from 0 to 5 for bushpigs.

### Selection of the Pigs, Collaring, and Data Collection

Within the study site (i.e., within farmland within a distance of 25 km from the MFNP boundary, see Figure 1), we selected 14 domestic pigs meeting the following criteria: they were kept free-range, their body size was large enough to fit with the harness adjustment (i.e., above 4 months) and they were owned by different families who agreed to the study. The number was also dictated by the number of GPS collars available. In addition, we planned to have a balanced sample between males and females, rainy and dry seasons and looked for a subsample of neighboring pigs (belonging to adjacent homesteads). We excluded sows being in the last trimester of pregnancy or those nursing piglets. Pigs that were due to be slaughtered in the next week were also excluded. Permission to carry out the study was granted by the Uganda National Council for Science and Technology under the reference number A497. A written consent from the District veterinary officer was obtained prior to the start of any activity in the area. At the time of the study, participants were informed that the study was voluntary, confidential, and that they had the choice of ending their participation at any time. An informed consent was given by all participants prior to the implementation of the study.

**Figure 2:**
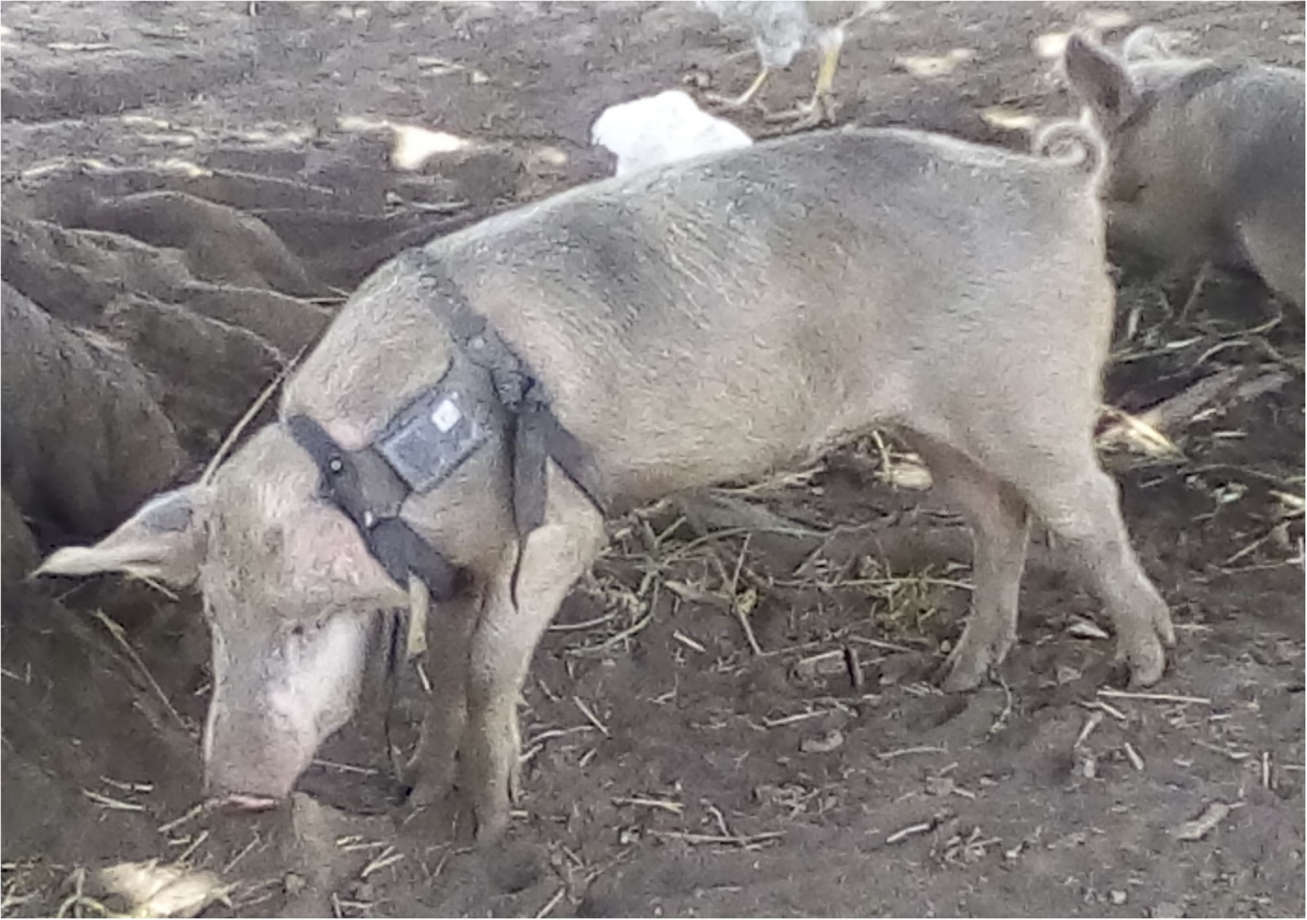
Domestic pig fitted with a GPS-GSM harness (Savannah Tracking, Ltd., Nairobi, Kenya).

The pigs were manually restrained and fitted with a GPS unit mounted on a harness (Savannah Tracking, Ltd., Nairobi, Kenya). The harnesses were made of straps which were not extensible (Figure 2). Elastic material could be more adapted to rapid growth of the body but it is also more fragile, that is why it was not used by the collar manufacturer. We used 7 GPS GSM and 7 GPS Iridium collars. Data were uploaded daily to a server through either GSM or iridium satellite transmission, depending on the type of collar used. Prior to deployment, the accuracy of the GPS was tested in a stationary position, under different vegetation covers with the program that was planned to be set when deployed on pigs. The maximum margin of error on the GPS locations provided by the collars was assessed to be 5 m and did not differ between the two transmission systems.

Collars were programmed to take one location every 30 min and were deployed for 2 weeks on each selected pig. We considered this schedule as the best compromise between precision, saving of battery power (some of the collars were to be deployed again or had already been deployed) and duration of the monitoring (i.e., we considered that 2 weeks of tracking were representative of the usual activity of the pig). To study the effect of the season, we monitored 6 pigs during the dry season and 4 pigs during the rainy season (Table 1).

**Table 1:** Characteristics of the collared pigs.

**Pig ID**	**Sex**	**Tracking duration (days)**	**Number of days used in the analysis**	**Number of nights used in the analysis**	**Number of locations**	**Months of tracking**	**Season**
1	M	13	12	13	306	March 2016	Dry
2	M	13	12	13	599	March-April 2016	Dry
3	F	17	13	11	544	April-May 2016	Rainy
4	M	7	6	5	226	June 2016	Rainy
5	F	5	4	5	207	October 2016	Rainy
6	F	9	9	9	416	October-November 2016	Rainy
7	F	14	12	13	578	January 2017	Dry
8	M	9	8	8	369	January 2017	Dry
9	M	11	10	11	504	March 2017	Dry
10	F	11	10	11	501	March-April 2017	Dry

### Home Range, Daily Distance, and Activity Pattern

To maximize the precision of the estimate, each home range was generated from all the available locations for each animal (26, 27). We obtained the home range and the core area using the fixed kernel method (28) taking into account 90% and 50% of the locations respectively, as it is commonly used in ecological studies (27, 29, 30). The smoothing parameter value was estimated by using the reference bandwidth method since the least-squares cross-validation method did not converge for most of the pigs (31, 32). The home range was additionally estimated by using the minimum convex polygon, taking into account 100% of the locations (100% MCP) in order to determine the maximum area the pigs were able to cover. These treatments were performed using the adehabitat R package (33). The areas of the home ranges and core areas were then calculated by using QGis 2.18 (34).

The full day distance for each pig was calculated by connecting the consecutive locations belonging to the same day, from 1 am to midnight (i.e., during 24 h). The first and last days of monitoring as well as the days with more than 10% of missing data (i.e., at least 5 missed locations) were excluded from the analysis. This was followed by computing the minimum, maximum and mean distances for each animal.

To determine the activity pattern, we split each full monitoring day into daytime (i.e., from 7 a.m. to 7 p.m.) (daily distance) and night time (i.e., from 7 p.m. to 7 a.m.) (nightly distance) and computed the minimum, maximum and mean distances for these 2 periods for each pig.

The maximum and mean distance from the individual homestead were calculated. To do this, the perimeter of the homestead, being that area utilized by the house for domestic activity (therefore excluding crop fields), was tracked by walking along the boundary using a handheld GPS unit (Garmin GPS Map 60Cx). Then, a polygon layer based on this perimeter corresponding to the homestead was generated with a buffer of 5 m to take into account the accuracy of the GPS units used. The centroid of this polygon was then created. We calculated the maximum, mean and standard deviation of the distance between this centroid and all the recorded locations except the ones falling under the homestead layer. All these distance calculations were made using QGis 2.18 (29).

We checked whether the home ranges (yielded by the kernel 90% and the MCP 100%), the full day, daily and nightly distances had a normal distribution using the Shapiro-Wilks test of normality. We then performed univariate analysis to check if these variables differed between the sex of the pigs, the season (dry vs. rainy) and the size of the herd (less or equal to 4 pigs vs. more than 4 pigs) using a Student test when the variables had a normal distribution and a Wilcoxon rank test in the other cases. We also compared the daily and nightly distances made by the pigs. These statistical analyses were performed with R version 3.4.2 (35).

### Use of Habitat

Once the pig tracking was completed and all the GPS locations retrieved, the GPS coordinates were plotted on a map and a landscape item was assigned to every location. This was done by going physically to the locations: for each location the GPS coordinates were entered in a handheld GPS unit (Garmin GPS Map 60Cx) enabling the operator to reach the locations uploaded from the pig's collar. The corresponding landscape was recorded according to one of these 5 categories: 1) homestead, 2) crop, 3) grassland (including also bush and forest), 4) waterpoint *(*river, borehole, pond, swamp or spring) and 5) road. When “homestead” was assigned, it was noted whether it was the one to which the pig belonged, or another one. The number of “other homesteads” was recorded.

For each pig, we used the same dataset as for the full day distances (see paragraph 2.3) i.e., where days with more than 10% of missing data were excluded. We calculated the use of each habitat item by the ratio: number of locations falling into the habitat item *i* to the total number of locations *n*.

As previous studies carried out in the same study sites pointed out that waterpoints and crops may be items at risk for interactions between domestic and wild pigs (22, 24), we focused on these two items by checking if their use was different between day and night and between the rainy and the dry seasons. We kept the definition of daytime and night time and used the same tests and software as for the activity pattern (see paragraph Home Range, Daily Distance, and Activity Pattern).

## Results

### Data Collected

Fourteen pigs were collared between March 2016 and April 2017. Among these 14, two lost their collars 2 days after deployment and two reported for only 1 or 2 days. For these reasons, data from these 4 pigs were excluded from the analysis.

Out of the 10 remaining pigs, five were females and five were males. Six were tracked during the dry season and four during the rainy season (Table 1 and Supplementary Material). Their age ranged between 5 and 9 months. The data regarding the use of habitat could not be collected for one pig (ID 10) within the time frame of the study. As a consequence, all the analysis regarding the use of habitat was performed on nine pigs.

The mean duration of the tracking was 10.7 days (range: 5–17 days). The deployment had to be shortened for several pigs, due to either a fast growth of the body size, leading to remove the collar earlier than planned to prevent injury (4 pigs), or a stop in the reporting (2 pigs) or the tethering of the pig (1 pig; despite the fact that the pig's owner agreed to take part in the study and leave his pig roaming freely, he had to tether the pig because it damaged lots of crops). The number of days and nights as well as the number of locations used for the analysis are shown in Table 1.

### Home Range, Daily Distance, and Activity Pattern

**Figure 3:**
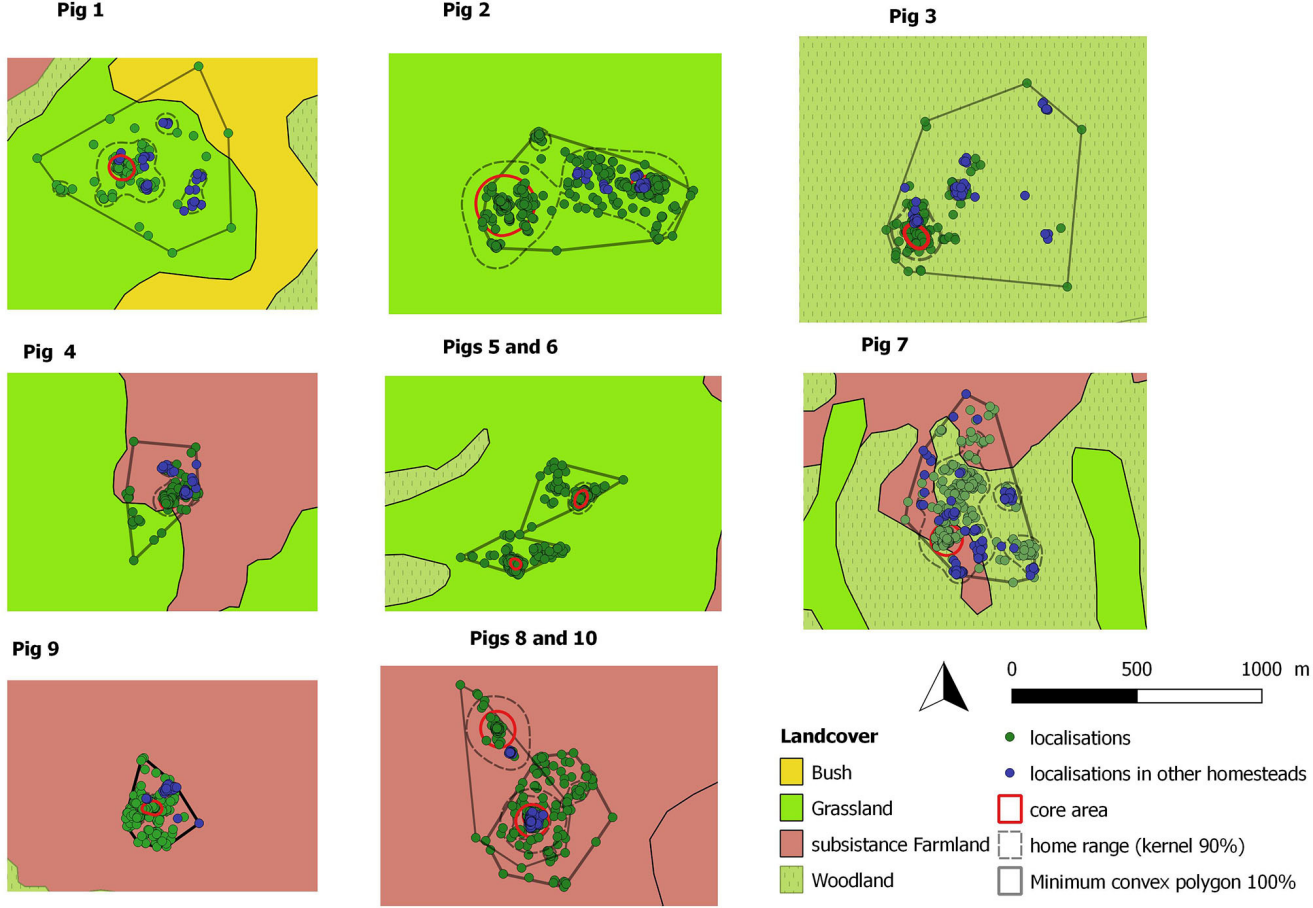
Collected locations, home ranges and core areas used by the 10 monitored pigs plotted on the different landcover types. Pigs 1, 2, 7, 8, 9, 10 were monitored during the dry season and pigs 3, 4, 5, 6 during the rainy season. The use of habitat was not studied for pig 10. The blue dots localized in his home range corresponds to pig 8's locations.

The pig home ranges varied between 8,078 and 253,327 m^2^, with an average of 74,113 m^2^. The maximum area covered by the monitored pigs, yielded by the MCP 100% varied between 35,965 and 475,077 m^2^. The size of the core area varied from 1,317 to 50,769 m^2^ (Figure 3 and Table 2). For every pig, the core area included the homestead where the pig belonged to and for two pigs, it also included another homestead (Figure 3).

**Table 2:** Details of the movements of the ten collared pigs.

**Pig ID**	**MCP 100% (m^2^)**	**Home range (m^2^)**	**Core area (m^2^)**	**Full day distance (m)[min-max], mean**	**Daily distance (m) [min-max], mean**	**Nightly distance (m)[min-max], mean**	**Distance to homestead (m) max mean**
1	342,703	76,570	7,647	[1,407–3,237], 2,168	[265–2,336], 1,198	[111–1,236], 553	512 94
2	280,395	253,327	50,769	[1,614–3,646] 2,574	[560–1,407], 872	[1,124–2,463], 1,570	760 300
3	475,077	38,014	6,669	[1,392–2,780], 1,899	[675–1,520], 1,089	[352–1,479], 726	787 131
4	93,771	28,644	4,935	[995–2,624], 1,843	[288–1,667], 932	[267–1,065], 563	311 87
5	50,884	15,452	2,540	[1,084–1,507], 1,339	[524–933], 816	[319–439], 396	297 71
6	35,965	8,078	1,317	[1,092–1,949], 1,461	[307–1,482], 975	[181–487],293	219 64
7	252,994	144,621	12,295	[2,106–3,246], 2,425	[207–603], 420	[1,520–26,83], 2,070	577 199
8	180,444	111,410	28,927	[272–3,153], 1,675	[130–2,453], 1,008	[142–791], 316	552 338
9	62,798	24,821	3,393	[1,839–2,937], 2,232	[1,312–1,949], 1,677	[171–653], 390	206 86
10	173,164	40,189	3,826	[1,353–2,647], 1,960	[692–1,350], 1,031	[432–1,107], 702	NA
Mean	194,820	74,113	12,232	1,958	1,002	758	152

**Figure 4:**
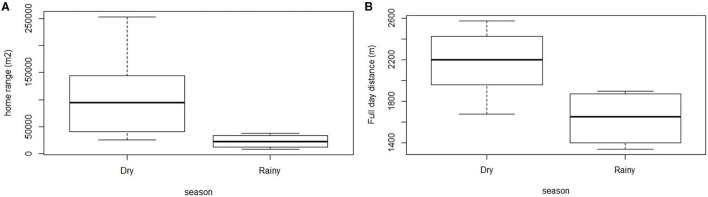
Distribution of the home range **(A)** and the mean 24 h distance **(B)** of the 10 monitored pigs according to the season. Intervals defining boxes represent the interquartile range (IQR), while intervals out of the boxes (whiskers) show the highest and lowest values within 1.5 × IQR.

Home ranges were significantly bigger during the dry season than during the rainy season (Wilcoxon test, *W* = 22, *p* = 0.04; Figure 4). The mean full day distance ranged from 420 to 1,677 m and was statistically longer in the dry season than in the rainy season (Student test, *t* = 2.7, *p* = 0.03; Figure 4). Mean values between the distances traveled during daytime and night time by each pig were higher for diurnal (1,002 m) than for nocturnal measures (758 m) but these differences were not significant. The monitored pigs roamed away from their homestead at a mean distance ranging from 64 to 338 m (Table 2). No significant difference was found for any of the measured indicators, neither between males and females nor according to the size of the herd.

### Use of Habitat

**Figure 5:**
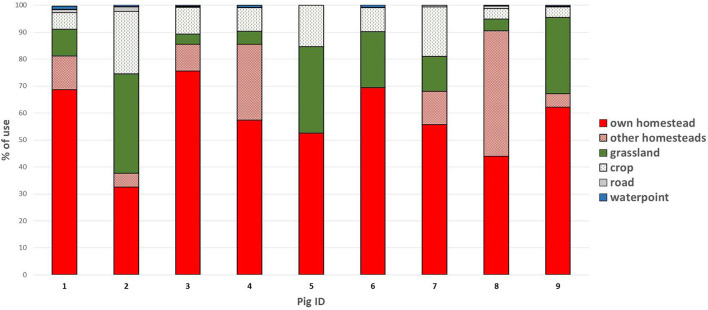
Use of the 6 habitats of interest by the 9 monitored domestic pigs, expressed in proportion of the total recorded locations.

The monitored pigs used mainly their own homestead except the pigs 2 and 8, which used mostly grassland and other homesteads, respectively. The mean use of other homesteads was 13.3% and the number of other homesteads visited by the pigs varied from 1 to 18 with an average of 6. The water points were the least used habitat accounting for a mean use of 0.5% and ranging between 0 and 1.3% (Figure 5). As the use of water points was very limited and equal to 0 for two pigs, we did not perform any test regarding this item. The use of crops ranged between 3.8 and 23.1% with an average of 10.9%. Cassava and maize fields were the most visited crops. No statistical difference was found between the two seasons and between day time and night time.

## Discussion

The use of spatial-temporal analysis in veterinary medicine has proven vital in understanding the epidemiology of animal and transboundary diseases across Africa, especially when dealing with free-ranging domestic and wild animals (36–38). In the context of Uganda where ASF is highly prevalent and the free-ranging pig farming system is highly widespread in rural areas, analyzing the spatiotemporal patterns of such pig systems is important to better understand the potential risks of disease exposure, transmission and possible consequences such as pig losses and reduced productivity.

Although ecological studies documenting the spatial dimension of domestic pig movements are scarce in the African context, one study addressed this topic in Kenya (29). However, this is the first time that this approach is implemented in the context of an ASFV infected area at the interface of a wildlife national park where the presence of a warthog—tick sylvatic cycle and the sympatric presence of warthogs and bushpigs with domestic pigs is well described (22, 24). The small number of pigs we had in our study limits the robustness of our results. Increasing the sample size would have enabled to gain power in the analysis and would have smoothed the possible individual variability that might have interfered with the effect of the variables tested at a population level. In the study carried out in Kenya, Thomas et al. (38) also monitored 10 free-ranging pigs, five being tracked during the dry season and five others during the rainy seasons and they did not find any significant effect of the season on the pigs' movements. In this case, pigs seemed to move larger average daily distances than in our study site (for instance around 4,000 vs. 1,000 m in our study). This emphasizes that movement of free-ranging pigs can differ from one rural settings to another, depending on the specificity of husbandry practices and the availability of food and water resources. Consequently, the results obtained in the context of such small-scale studies should not be extrapolated to other areas.

Our study reveals that the free ranging domestic pig home ranges were significantly larger during the dry season than during the rainy season, with mean full day distances statistically longer in the dry season than in the rainy season. Coincidentally, this is also the season where the number of reported ASF cases is higher in the study area and considered as a risk factor for ASF outbreaks (11, 16, 22). The large home ranges used by pigs in the dry season, combined with higher number of pigs kept free-range rather than tethered or confined may increase contacts between infected domestic pigs, fomites or carcasses from different infected farms.

Incursions into other homesteads were quite frequent in our study (representing 13% of the habitat use), though variable among pigs (seven pigs were frequenting an average of six other homesteads). Considering that the infectious period can last between 5 and 14 days (39) and even beyond in the case of pigs which have survived the acute phase of the disease [25 days, (40)], the probability of excreting ASF virus and thus representing a source of infection for other domestic and wild pigs is non-negligible.

As a result, these movements could potentially contribute to ASF transmission between households in case of an outbreak. In addition, the dry season is the most productive season in terms of wild pig hunting (22), which makes potential exposure to wild pig hunting leftovers more likely than during the rainy season. Finally, yet importantly, the dry season is likely to attract wild and domestic pigs around water points, as suggested by interviews with farmers in a previous study (22). However, surprisingly, our results showed a very limited dependence of domestic pig movements on water points. A possible explanation could be that water is found within the homesteads or in puddles which we did not assign as water point. We did not record wether pigs were provided with water or/and with feed, which could also have influenced the dependence of the pigs on water points and possibly to crops. Crops represented more than 10% of the habitat used by the domestic pigs, most of them being cassava and maize. This result is not surprising given that these cereals are very palatable for pigs, which are free to feed in cultivated land and are often underfed by their owners. Payne et al. (24) reported that cassava is particularly sought after during the night by the bushpigs in this area. In our study, we found that domestic pigs moved also during the night making this type of habitat a potential hotspot for interactions between this two sympatric species and therefore, for ASF transmission.

Regardless of the season, an additional permanent source of virus in the context of the study area is the probability for a free roaming domestic pig to become exposed to an ASF infected soft tick (*Ornithodoros moubata* complex) bite. Despite the fact that there are no data on the rate of infestation of warthog burrows with ASF-infected *O.moubata* in the study area, soft ticks infected with ASFV have been found and the occurrence of a sylvatic cycle in MFNP has been confirmed (unpublished results). Therefore, the likelihood of exposure to this permanent source of virus in this environment cannot be ignored. At the interface of MFNP, sightings of warthogs have been reported up to 25 km from the park boundary (22) into the domestic homesteads. The same study reports that almost ¼ of the farmers interviewed in our study area had observed active warthog burrows in proximity and in 60% of cases at <3 km from their homesteads. This confirms that domestic pigs kept in this area, like the ones we selected in our study, are sharing the space and resources with warthogs and their ticks. Coincidentally, a recent study assessing potential wild-domestic pig interactions at the interface of wildlife game reserve in South Africa, also reported that local pig farmers reported wild pig sightings up to 25 km from the boundary of the reserve (41). This measure of the potential home range of wild pigs outside of natural reserves is only based on interviews and should be confirmed by more precise ecological study methods. Nevertheless, it provides a similar indication of the spatial overlap between wild and domestic pigs at the wildlife-livestock interface of two different protected areas in East and Southern Africa.

Seven out of nine pigs makes 78% of pigs visiting other homesteads in our sample. Assuming this percentage is right, and considering there are a total of 12,800 pigs estimated in the area, there would be nearly 10,000 pigs visiting other homesteads per year. In case of an outbreak of ASF, the impact of these movements in disease dissemination is far from being negligible. Similarly, wild and domestic pigs in tropical areas can carry several infectious and parasitic diseases such as cysticercosis, trichinellosis, toxoplasmosis, porcine circovirus or *Actinobacillus pleuropneumoniae* (2, 3, 9, 29, 42). In this context, the potential high contact rate between pigs from different herds, exposes them to higher probabilities of disease exposure and transmission to other susceptible individuals.

This study provides additional evidence of the high risk faced by pig farming lacking basic biosecurity measures at the interface of a protected area in East Africa. Further studies should target at quantifying that risk by assessing wild and domestic pig densities and identifying potential contacts in hotspot interaction locations. Contact networks could be drawn from our data, enabling to better assess the connectivity between domestic pigs from different homesteads and map the risk of transmission of diseases such as ASF at fine-scale (43). Our findings could further be used to target effective preventive measures aiming to mitigate disease transmission risks in low biosecurity farming systems. As an alternative, pig farming with simple and affordable but efficient measures of higher biosecurity should be promoted in the area with the goal to inform on the advantages that could be found in terms of higher productivity and profit. For example, community-based animal health workers could be involved in designing local scale homestead disease prevention strategies. However, awareness on the availability of biosecurity and control measures does not guarantee their implementation (11). Indeed, the adoption of disease prevention and biosecurity measures among small scale farmers in poor resource settings such as the interface of MFNP, is far from complete, mainly due to financial constraints, despite acknowledging the capacity of biosecurity to protect pigs from ASF (44) and other diseases. While the vaccine is still awaited, another interesting alternative unexplored to date, could be exploitation of innate resistance to the virus, which is fully effective in wild African suids and has been observed in some domestic pig populations in areas of prolonged endemicity (14).

## Data Availability Statement

The original contributions presented in the study are included in the article/Supplementary Material, further inquiries can be directed to the corresponding author/s.

## Ethics Statement

The animal study was reviewed and approved by Uganda National Council for Science and Technology under the reference number A497. A written consent from the District veterinary officer was obtained prior to the start of any activity in the area. Written informed consent was obtained from the owners for the participation of their animals in this study.

## Author Contributions

AP and PO collected the data. AP performed the analysis. AP, KS, CM, and FJ conceptualized the thrust and focus of the manuscript. All authors participated in drafting the manuscript or revising it critically for content. All authors were involved in the study design.

## Funding

This study was financed by APHIS (US Department of Agriculture, Animal and Plant Health Inspection Service, APHIS Agreement No. 13-7440-0989-GR) Wellcome trust (Grant 105684/Z/14/Z) and French Embassy in Uganda (Convention de subvention 10/10/2016. Appui CIRAD 185UGA0079).

## Conflict of Interest

The authors declare that the research was conducted in the absence of any commercial or financial relationships that could be construed as a potential conflict of interest.

## Publisher's Note

All claims expressed in this article are solely those of the authors and do not necessarily represent those of their affiliated organizations, or those of the publisher, the editors and the reviewers. Any product that may be evaluated in this article, or claim that may be made by its manufacturer, is not guaranteed or endorsed by the publisher.

## Supplementary Material

Supplementary Table 1 GPS data file.
